# Using stakeholders' preference for ecosystems and ecosystem services as an economic basis underlying strategic conservation planning

**DOI:** 10.1016/j.heliyon.2020.e05827

**Published:** 2020-12-23

**Authors:** J. Carl Ureta, Michael Vassalos, Marzieh Motallebi, Robert Baldwin, Joan Ureta

**Affiliations:** a Forestry and Environmental Conservation Dept., Clemson University, Clemson, South Carolina, USA; b Department of Agricultural Sciences, Clemson University, Clemson, South Carolina, USA; c Forestry and Environmental Conservation Dept., Baruch Institute of Coastal Ecology and Forest Science, Clemson University, Georgetown, South Carolina, USA

**Keywords:** Ecosystems ranking, Ecosystem services ranking, Stakeholder approach, Conservation planning, Preference analysis, Ranking analysis

## Abstract

Ecosystem services (ES), commonly defined as the benefits people get from ecosystems, are key components in improving human well-being. However, as land utilization transitions from forest and agricultural land to urban areas and industrial complexes, the continuous provision of ES is affected. To ensure sustainable development, conservation programs should be implemented that consider both the stakeholders' well-being while also maintaining ecosystem health and integrity. Consequently, to improve the strategic implementation of conservation programs, it is critical to understand stakeholders' preferences.

Using an online survey, we elicited South Carolina residents' preference in prioritizing the target ecosystems and ecosystem services in the state. The results identified that the priority ecosystem service is water quality regulation. However, the residents' preference for water quality regulation does not discredit the importance of maintaining the continuous water supply provision. In terms of ecosystem preference, residents indicated that the forest ecosystem is the priority ecosystem to be conserved, particularly for younger residents, respondents with high income, and those in the midland and the upstate. This could be attributed to the forest's effect on the ecosystem services that these respondents receive, particularly towards water-related ecosystem services. Understanding the residents' preference provides information that could improve the state water plans and other potential policy implications to land use-land cover planning and landscape sustainability management.

## 1 Introduction

Ecosystems services (ES), commonly defined as material and non-material benefits that people receive from the environment (Millenium Ecosystem Assessment, 2005), affect the economy and eventually improve society's well-being. Specifically, the provision of ES directly improve society's well-being in five dimensions: (1) basic material for a good life, (2) freedom of choice, (3) health, (4) good social relations, and (5) security (Millenium Ecosystem Assessment, 2005; Wu 2013; United Nations, 2014).

While ES improve societal well-being, their continuous provision is directly dependent on the ecosystem's health and integrity. This reciprocal relationship is the fundamental basis of Social-Ecological Systems (SES) or coupled human-environment systems (CHES) (Wu, 2013). Social-ecological systems' components are focused on people and other organisms using the ecosystem services as the main linkage. This complex system is not merely a summation of “social” and “ecological” systems, as it develops numerous unique characteristics commonly referred to as emergent properties (Cumming, 2011). Since SES are systems of people and nature, it follows that humans should be seen as part of nature, and nature should be seen as part of society (Berkes and Folke, 2000). The SES framework may be central in pursuing sustainability and resiliency across the landscape. While the goal of sustainability is centered on improving human well-being (Brundtland, 1987), this cannot be achieved without protecting ecosystems (Wu, 2013). Therefore, new methodologies that integrate both the social and ecological aspects are being explored, such as the ES-based approach (Asah et al., 2012; Díaz et al., 2015; Raum, 2018).

The basic foundation of the ES-based approach is that human and ecological well-being are tightly connected to the sustainable management of resources (Tallis and Polasky, 2009). Apart from the improvement to the chemical and biophysical characteristics of an ecosystem, the ES-based approach measures the effectiveness of interventions and programs by considering the benefits that stakeholders derive from the ecosystem. Notably, improvement of human well-being is a core principle for an ES approach (Millenium Ecosystem Assessment, 2005). Consequently, scholars have focused on evaluating consumers' preferences and welfare impact from the changes of ES.

Improvement of ES provides a variety of societal benefits. For instance, the application of green spaces and green infrastructures improves the urban environment while also contributing to flood mitigation, water quality improvement, and microclimate provision. These regulating ecosystem services contribute to human health by lowering human exposure to contaminated floodwaters, removing toxicants, trapping contaminants, and mitigating extreme temperatures (Summers et al., 2018). Socio-cultural ecosystem services also provide multiple benefits, such as therapeutic benefits and heritage benefits (Schmidt et al., 2016). Besides, since one ecosystem service typically has a synergistic effect with other ecosystem services, the impacts of the socio-cultural ecosystem services also affect other types of ecosystem services (Schmidt et al., 2016).

In contrast, land-use changes that favor urban expansion and industrialization negatively affect ecosystems, resulting in degradation and decline of ecosystem services (Liu et al., 2012; Huang et al., 2013; Krkoška lorencová et al., 2016; Motoshita et al., 2016). While it has a negative impact on human well-being, the decline of the ecosystem services is being overshadowed by the potential economic gains of these land-use changes. To assess if the ES's foregone benefit is comparable to the economic gains, several indexes and metrics have been utilized in the literature (Wainger and Mazzotta, 2011; Schmidt et al., 2016; Leviston et al., 2018; Olander et al., 2018). Although these metrics provide a general understanding of ecosystem services benefits, identifying which particular social benefit is still not commonly understood (Schmidt et al., 2016).

Other research endeavors focus on evaluating residents' Willingness to Pay (WTP) to support water quality improvement (Calderon et al., 2012; Doherty et al., 2014; Ureta et al., 2016; Khan and Zhao, 2019). A consensus across these studies is that residents have a higher willingness to pay (WTP) for good water quality (Doherty et al., 2014; Khan et al., 2019) and prefer water quality improvements more than water distribution (Khan and Zhao, 2019). However, divergence exists. For example, studies at the local scale also show that habitat and recreational ecosystem services are valued more in certain areas (Castro et al., 2016). A study in the United States found that there is a homogeneous distribution of WTP for the improvement of water quality across the nation. At the same time, it may vary across different geographic locations for other ecosystem services (Aguilar et al., 2018). Furthermore, residents who are willing to preserve the environmental quality within the watershed typically relate their WTP to water quality improvement (Brox et al., 1996). Although literatures regarding understanding stakeholder preference are available mainly on the topic of willingness-to-pay and welfare economics, there is limited research, particularly on ecosystem and ecosystem service preference used for conservation planning at a state level. Furthermore, WTP approaches are prone to large confidence intervals which gives plenty of room for uncertainty (Brent et al., 2017). While WTP estimates are useful information for policy-makers, it should only be one of the multiple inputs to be considered (Brent et al., 2017). Valuation methods may provide a definitive and robust case to consider the ecosystem services in the decision-making process, but it is essential to understand the limitations. It does not entirely capture the full values for many non-use services, and the estimated values are often non-transferable to other sites since no market is involved (Wainger and Mazzotta, 2011). Hence, other approaches to decision-making need to be considered. Qualitative accounts, multi-criteria methods, and preferential ranking analysis also provide a different perspective in understanding people's perception in the social and environmental context (Goldman et al., 2007; Thompson, 2018). Since the relationship between people's perception and their social-environmental context is complex, this highlights the importance of considering perception in crafting more effective and inclusive landscape management strategies (Quintas-Soriano et al., 2018). The use of stakeholder preference and perception is effective in formulating policies for ecosystem service and natural resource conservation (Quintas-Soriano et al., 2018) and as a guide for modeling and management efforts (Elwell et al., 2018).

Despite the growing interest in adopting an ES-based management approach (Daily et al., 2009), their implementation is challenging for several reasons. Land managers should have the capacity to perform ES analyses and the statutory authority over the land to conduct these approaches (Noe et al., 2017). Furthermore, practitioners of ES-based management approaches also have to have the legal mandate to integrate ES in their analyses (Presnall et al., 2015). Also, even with strong statutory support, an unclear understanding of the concept of ES among stakeholders limits the capacity to perform ES-based analyses (Sitas et al., 2014). In the Southern US, since the majority of forest area is privately owned (Butler and Wear, 2013; South Carolina Forestry Commission, 2015), implementation of ES is even more challenging. For example, landowners need to voluntarily implement the intervention. Otherwise, an incentive mechanism has to be developed to attract landowners. One way to address these challenges is by doing a “bottom-up” stakeholder-based approach to tailor-fit programs based on the stakeholders' preference and perception (Ernst and van Riemsdijk, 2013; Ricart Casadevall, 2016; Raum, 2018; Song and Hu, 2019; Zoderer et al., 2019).

Stakeholders'^1^ perceptions play an important role in strategically selecting interventions (Asah et al., 2012; Raum, 2018). The literature on assessing water users' perspectives typically focuses on large groups and intermediate consumers, such as farmers and landowners. Although this approach has provided significant insights and advanced stakeholder involvement for selecting interventions, the final stakeholder recipients of ES (typically household residents) are less often consulted regarding their preference (Pellett and Walker, 2018; Quintas-Soriano et al., 2018; Ricart et al., 2018; Tumpach et al., 2018; Khan and Zhao, 2019). Not accounting for the residents' perspective as the final recipient of the ES could result in a misalignment in the implementation of policies for conservation.

This study is an effort to fill this gap in the literature. Specifically, we aim to examine South Carolina (SC) residents' preferences for what type of ecosystem and which ecosystem service should be targeted for the implementation of conservation programs. Furthermore, we evaluate the factors that affect their preferences.

South Carolina is selected for several reasons. First, the state of South Carolina has abundant surface water sources. This is due to the state's geographic location, topography, and natural land cover. Seventy percent (70%) of the state's water source comes from the rivers and streams, while groundwater provides 30% of the SC water sources (US Environmental Protection Agency, 2013). The surface water source is more convenient to access, resulting in a more efficient distribution of water. Even with abundant water resources, SC is crafting water policies and plans ensuring the continuous provision of water supply to meet with the expected demand (Harder et al., 2020; Hargrove and Heyman, 2020). In 2008, South Carolina experienced the worst drought that the state has recorded (US Environmental Protection Agency, 2013). Furthermore, the state population is projected to increase by 18% from 2010 to 2030. This plan focuses explicitly on regulating water supply and water consumption by monitoring and implementing regulatory programs. Moreover, as the 2014 state water plans are updated (South Carolina Dept. of Natural Resources, 2020), water resource managers are interested in knowing the perception of South Carolinians towards the state of the environment. Lastly, people's preferences can impact funding allocations for conservation programs.

Despite the importance of the state's ecosystem services, to the best of our knowledge, there is no study that has evaluated the residents' preference for conserving these services. Following the previous studies that linked the residents' preference to prioritizing water quality improvement as an ecosystem service (Brox et al., 1996; Doherty et al., 2014; Castro et al., 2016; Aguilar et al., 2018; Khan et al., 2019; Khan and Zhao, 2019), we hypothesized that South Carolina residents would prefer improved water quality over the other ecosystem services examined.

However, while water supply is critically important, other aspects of the ecosystem services such as water quality improvement could be left unchecked. Due to the continuously changing land use-land cover (LULC) from increased urban and residential areas, water quality is affected through an increase in non-point source pollution, unsustainable agricultural activities, urbanization, forest degradation, and landscape fragmentation (Huang et al., 2013; Abas and Hashim, 2014; Kaushal et al., 2017; Camara et al., 2019). Therefore, national and state-level interventions such as the Environmental Quality Incentives Program (EQIP), the National Water Quality Initiative (NWQI) of the United States Department of Agriculture – Natural Resources Conservation Service, and establishment of conservation easements were developed to encourage sustainable practices and implementing conservation interventions for landowners.

Furthermore, while state water plans are focused on regulating water supply and demand, the determination of values and what to measure as the value of an ecosystem are subjective interpretations and can be arbitrary (Spangenberg and Settele, 2010). Furthermore, using economic pricing as a key valuation neglects other ways to understand ecosystem science (Norgaard, 2010). Therefore, using purely economic lenses does not provide a holistic understanding of ecosystems and their services (Kosoy and Corbera, 2010). This paper looks at another perspective on the strategic implementation of conservation programs by understanding the residents' preference as the final recipients of the ecosystem services.

## 2 Methodology

### 2.1 Study site – data collection

South Carolina lies in the Southeast Region of the US, with approximately 83,000 km^2^ land area. The majority of its land use is composed of forest land (36%)^2^, pasture and agricultural land (30%) (US Geological Survey (USGS) Gap Analysis Project (GAP), 2012). The state is home to almost five million people. Manufacturing, finance, and real estate industries are the leading contributors to the state economy (US Bureau of Economic Analysis). Nevertheless, the agribusiness industry contributed $982 million to the state's Gross Domestic Product (GDP).

**Figure 1:**
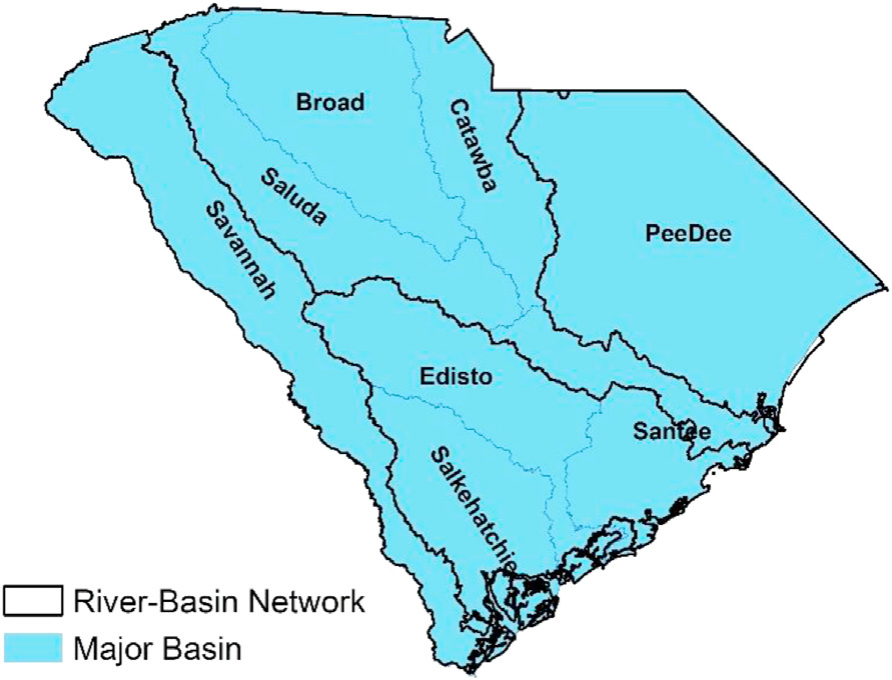
South Carolina river basin networks.

South Carolina has four river-basin networks (Figure 1): Savannah, Edisto-Salkehatchie, Santee, and Pee Dee. These networks are further subdivided into eight major basins (Bureau of Water, 2008). These major basins hold an intricate network of streams and rivers which provide essential ecosystem services such as water supply, water quality regulation, recreational activities, wildlife habitat, and hydropower provision.

To understand the stakeholders' perception towards ecosystems and their services within the state, we surveyed 1500 households across SC using the online survey platform Qualtrics in 2019. The online survey was utilized as data collection because, as of 2017, most SC residents (79%) have access to the internet (U.S. Department of Commerce Census Bureau, 2019). A simple random sampling technique from the list of residents' emails was used to select the survey respondents. To ensure representation from different counties, the number of samples taken was considered in proportion to the county population.

Furthermore, to consider the household's geographic location, the zip codes provided by the respondents were used to calculate the centroid of the zip code area through ArcGIS. This became a representation of the respondents' household location. The geographic location was used to correlate the respondents' characteristics and the proximity of their household to nearby environmental attributes such as stream quality and the presence of protected areas.

### 2.2 Survey design

The survey instrument consisted of four sections. Section 1 elicited baseline information about the respondents' understanding of the concepts of ecosystem and ecosystem services. This section also elicited the respondents' satisfaction rating towards the current state of the ecosystem within their vicinities, such as the general impression of the streams and households' water quality, the quality of air, the amount of water that they can access, and the overall impression to the quality of the environment in the area. The second section was an infographic of the terminologies and concepts that were used throughout the survey. This ensures that respondents have a similar understanding of the research questions. Section 3 elicits their preference to priority ecosystem and ecosystem services. Provided with a list, respondents were asked to rank the listed ecosystem and ecosystem services, according to their prioritization, highlighting that funds for conservation programs are limited. The last section included questions about demographic characteristics.

The survey instrument was pre-tested with 32 residents of SC. The pre-testing evaluated the initial knowledge and perception of the respondents towards ecosystem services and conservation programs. Also, the pre-testing provided final inputs to the list of the commonly known ecosystem and ecosystem services in the area resulting in a more robust questionnaire specifically designed for South Carolina residents. Lastly, the pre-testing evaluated the wording, timing, etc., of the survey instrument.

The survey instrument was reviewed and approved by the Clemson University Institutional Review Board (IRB) to ensure that ethical guidelines on research activities involving human subjects are followed. The IRB approval number is IRB2018 – 139.

### 2.3 Analysis of ranked preference

Respondents were provided a list of ecosystems and ecosystem services as their options to choose from. The list was based on a focus group discussion workshop conducted as part of the study's preliminary activities. Each respondent was asked to rank the options according to their preferred prioritization, considering a limited implementation budget for conservation programs to improve or enhance these services. This method was also done for different ecosystems to elicit the priority ecosystems according to respondents' preferences (Table 1).

**Table 1:** List of ecosystems and ecosystem services for ranking.

List of Ecosystems	List of Ecosystem Services
• forests	• water quality (water quality regulation)
• rivers/lakes	• water supply (abundance of accessible water)
• farm/agricultural land	• air quality (air quality regulation such as carbon sequestration, filtering air pollution)
• wetland/marshes	• wildlife and habitat conservation
• mountain	• tourism and recreation (such as biking, walking, trail hiking)
• coastal plains/beaches	• heritage and cultural site importance
• ecosystems with recreational function	• hunting activities
	• fishing activities

Henry Garrett's ranking technique was utilized to analyze the overall ranked preference. Garrett's ranking technique is used primarily to determine the collective rank of options using a score value (Arunkaumar et al., 2018; Dhanavandan, 2016; Sedaghat, 2011). The ranking technique begins with estimating the percent position score of the options using the equation:

(1) $$\text{Percent}\;{\text{position}}_{\text{i}}=\frac{100\left(R_{ij}-0.5\right)}{N_{j}}$$

Where:

R_ij_ = Rank given for the *i*^th^ option by the *j*^th^ respondent
N_j_ = Number of variables ranked by the *j*^th^ respondent

The percent position estimated is converted through Garrett's table (Appendix 1) to determine the total rank score of the *i*^th^ option. The rank scores for each option *i* are added to get the overall value of scores. Eventually, the mean value of scores is calculated by dividing the overall rank scores by the number of respondents. The mean value of scores is ranked highest to lowest to determine the hierarchy of options (Dhanavandan, 2016).

Eq. (1) was used to all ranked attributes in the study – the rank of priority ecosystem and the rank of priority ES. In this manner, the resulting order identifies the priority ecosystem and ES of the respondents.

### 2.4 Understanding the respondents' preference

A maximum likelihood regression analysis was used to examine the respondents' characteristics that could have a statistically significant effect on their top-ranked options. Furthermore, the regression analysis provides information on which among the options will respondents likely select based on their differing characteristics. Since the nature of the dependent variable is categorical, the likelihood model that was used is a Multinomial Logistic regression or Multi-Logit regression (Greene, 1980).

The Multi-Logit regression is a non-linear regression that deals with multiple categorical situations. The model assumes that the options presented to a decision-maker are mutually exclusive, hence not correlated with each other. The model estimates the likelihood of choosing one option over other options. Because the options among the ecosystems and ecosystem services are unordered, the Multi-Logit model analyzes the primary question of “What is the respondent's priority ecosystem and ES among the list of options?”. The Multi-Logit regression analyzes if a specific option is “more or less preferred” in comparison to another option. The equation of the regression is as follows:

(2) $$P_{\text{ij}}=\frac{e^{\sum_{j=1}^{K}\alpha +\beta_{kj}X_{kji}}}{\sum_{j=1}^{K}e^{\sum_{j=1}^{K}\alpha +\beta_{kj}X_{kji}}}$$

Where *P*_*ij*_ is the estimated likelihood of choosing the option *j* for respondent *i*, or in the case of the study, the priority ecosystem or ES of respondent *i*. Furthermore, the numerator and the denominator depict the odds ratio of the chosen option in comparison to others. With *α* as a constant coefficient while *β*_*kj*_ is a vector of coefficients corresponding to the vector of attributes *X*_*kji*_. Attribute *X* could be any characteristic or attributes that have a significant contribution to the respondent's decision. The list of attributes used in the models is summarized in Table 2.

**Table 2:** Summary of attributes in the Multi-Logit model.

Attribute	Description	Levels
Endogenous variables		
• Priority ecosystem	the ecosystem which respondent ranked as 1st priority in the ranking analysis	1 - forest; 2 - rivers/lakes; 3 - farm/agricultural land; 4 - others
• Priority ecosystem service	the ecosystem service which respondent ranked as 1st priority in the ranking analysis	1 - water quality; 2 - water supply; 3 - other ES
Exogenous variables		
• Satisfaction rating to the overall quality of water	5-point Likert scale response to the perceived satisfaction towards the current water quality in the area, reclassified into two levels	1 - satisfied; 0 - otherwise
• Satisfaction rating to the abundance or amount of water accessible to the household	5-point Likert scale response to the perceived satisfaction towards the current water quality in the area, reclassified into two levels	1 - satisfied; 0 - otherwise
• Satisfaction rating to the overall state of the environment in the area	5-point Likert scale response to the perceived satisfaction towards the current water quality in the area, reclassified into two levels	1 - satisfied; 0 - otherwise
• Age	age of the respondent	year
• Income bracket	overall income category of the household	1 - less than $10,000 - $49,999; 2 - $50,000 - $99,999; 3 - more than $100,000
• Distance to an impaired stream	the proximity of the zip code centroid to the nearest impaired stream	meters
• Distance to a good water body	the proximity of the zip code centroid to the nearest good water body	meters
• Distance to a protected area	the proximity of the zip code centroid to the nearest protected area	meters
• Respondents' residential region	Geographic region of the respondents' residence	1 – low country/coastal; 2 – midland; 3 - upstate

The satisfaction rating and preferences towards an ecosystem or ecosystem service were included in the exogenous variables. To simplify the categories as inputs to the regression model, the satisfaction rating was consolidated and reclassified into a dummy variable, taking a value of 1 (satisfied) or 0 (otherwise). Respondents who answered 4 or 5 in their satisfaction rating was reclassified into 1, while the other ratings were classified into 0.

Following previous studies linking the demographic factors and its influence on environmental values and preferences (Abdul-Wahab and Abdo, 2010; Mangiafico et al., 2012; Leviston et al., 2018; Olander et al., 2018), demographic variables are included in the model. Furthermore, socio-economic characteristics are typical factors used in evaluating decision-making as this constitutes constraint attributes to respondents. This is typical to valuation and stakeholder involvement studies (Mangiafico et al., 2012; Marsh, 2014; Ureta et al., 2016; Seriño et al., 2017; Small et al., 2017; Soley et al., 2019).

Proximity to monitored ecosystems was included to represent a possible distance-effect of the quality of these ecosystems to the preference of the respondents. Monitored ecosystems are ecosystems that are regularly monitored and managed by authorities or landowners as indicators for environmental health. For this study, we focused on impaired streams - streams that did not meet the water quality standards and at least not open for public access due to water quality issues; water bodies such as lakes, large rivers, and ponds that are evaluated as with good quality (Bureau of Water, 2008; Bureau of Water, 2011; South Carolina Department of Health and Environmental Control, 2018); and protected areas - public and privately protected lands which were classified by US Geological Survey (USGS) through the Protected Area Database of the United States (PAD-US) (US Geological Survey (USGS) Gap Analysis Project (GAP), 2012) and a privately monitored dataset of The Nature Conservancy (TNC) (personal communication, 2019) in South Carolina. The proximity from monitored ecosystems could affect the respondent's preference since areas that could provide prime ecosystem services may not be equally distributed across the landscape (Watts et al., 2017; Lin et al., 2019). The proximities affect the stakeholders' preference as feedback of the impression of the quality of the nearby ecosystem (Weaver and Lawton, 2008), while the quality of the ecosystems can be associated with possible interventions.

### 2.5 Assessing the satisfaction rating of respondents

The respondents' impression of the general state of their environment was also elicited using a 5-point Likert scale satisfaction rating (1 being the lowest and 5 being the highest). Survey participants were asked four general questions about aspects of the environment: (1) satisfaction with the overall quality of water, (2) satisfaction with the abundance or amount of water accessible to their household, (3) satisfaction with the quality of air within their area, and (4) satisfaction with the overall state of the environment in their area. This question could serve as a feedback mechanism for conservation managers and professionals to understand how residents perceive the current state of the ecosystem and ecosystem services. This could indicate their awareness on the state of the environment and influence their preference for deciding which ecosystem and ecosystem services should be prioritized.

Since the respondents' satisfaction rating is highly localized, for visualization purposes, the satisfaction rating of each respondent was averaged per county to represent the overall mean satisfaction rating within the county. This captures the heterogeneity of the respondents' perceptions across the state. Furthermore, the county satisfaction ratings' median was utilized to measure the central tendency of the overall satisfaction per environmental attribute.

## 3 Results and discussion

### 3.1 Demographic characteristics

The demographic characteristics of the sample are reported in Table 3. The respondent demographics were compared to the state and national statistics to determine if the characteristics are representative of the population.

**Table 3:** Summary of the respondents' demographic profile.

Demographic characteristic	Study	SC	US
Median Age	47.3	39.7	38.2
Mean length of residency	22		
Mean Household size	2.77	2.57	2.63
Respondent gender			
Male	25%		
Female	75%		
Educational attainment			
Less than high school graduate	4%		
High school graduate (includes equivalency)	23%		
Some college or associate degree	38%		
Bachelor's degree or higher	35%	27%	31%
Employment status			
Employed	47%	56%	60%
Unemployed	25%	3%	3%
Retired	25%	40%	37%
Students	3%		
Income distribution			
Less than $10,000	9%	8%	6%
10k to 50k	44%	40%	35%
50k to 100k	33%	31%	30%
100k to 150k	11%	12%	15%
more than 150k	5%	9%	14%

Source: (United States Census Bureau, 2019).

Results show that, in terms of educational attainment, most of the respondents have some college degree. Furthermore, the number of respondents with a bachelor's degree or higher resembles the statistics of the state and national population. In terms of annual household income, the sampling distribution is closely similar to the state and national household income distribution. Overall, the results of the demographic characteristics indicate that the sampled respondents represent the demographic characteristics of the population.

The high frequency of unemployed respondents is not uncommon in online surveys since they can use online surveys as an extra income source (Smith et al., 2016; Ford, 2017). Furthermore, the gender imbalance of respondents is a common occurrence particularly in survey-based studies, since female household decision-makers tend to stay and manages the household (Calderon et al., 2012; Ureta et al. 2014, 2016). Moreover, studies showed that the participation rate of female respondents is higher for mail-in and online platforms due to the differences of female and male values operating in a gendered online environment (Smith, 2008; Mulder and de Bruijne, 2019).

### 3.2 Residents' impression of the current state of the environment

The results of the satisfaction ratings are reported in Table 4. Results showed that survey participants have the highest satisfaction rating in water supply characteristic followed by the air quality, while the water quality characteristic and the overall quality of the environment yielded the lowest rating with a mean rating that is not significantly different from each other. While it is not clear whether there is a connection between the water quality and the perception of the overall quality of the environment, this merits further investigation to understand the driving variables of their satisfaction rating.

**Table 4:** Summary of residents' satisfaction rating.

Attribute	Satisfaction rating (1 - lowest, 5 - highest)					Mean	*t*-test		
	Extremely dissatisfied (1)	Somewhat dissatisfied (2)	Neither satisfied nor dissatisfied (3)	Somewhat satisfied (4)	Extremely satisfied (5)		WQ	WS	AQ
Water quality (WQ)	63 (4%)	169 (11%)	221 (14%)	634 (41%)	468 (30%)	3.9			
Water supply (WS)	30 (2%)	49 (3%)	157 (10%)	447 (29%)	872 (56%)	4.3	0.000		
Air quality (AQ)	30 (2%)	109 (7%)	216 (14%)	654 (42%)	546 (35%)	4.1	0.000	0.000	
Overall quality of the environment	42 (3%)	157 (10%)	258 (17%)	726 (47%)	372 (24%)	3.8	0.107	0.000	0.000

**Figure 2:**
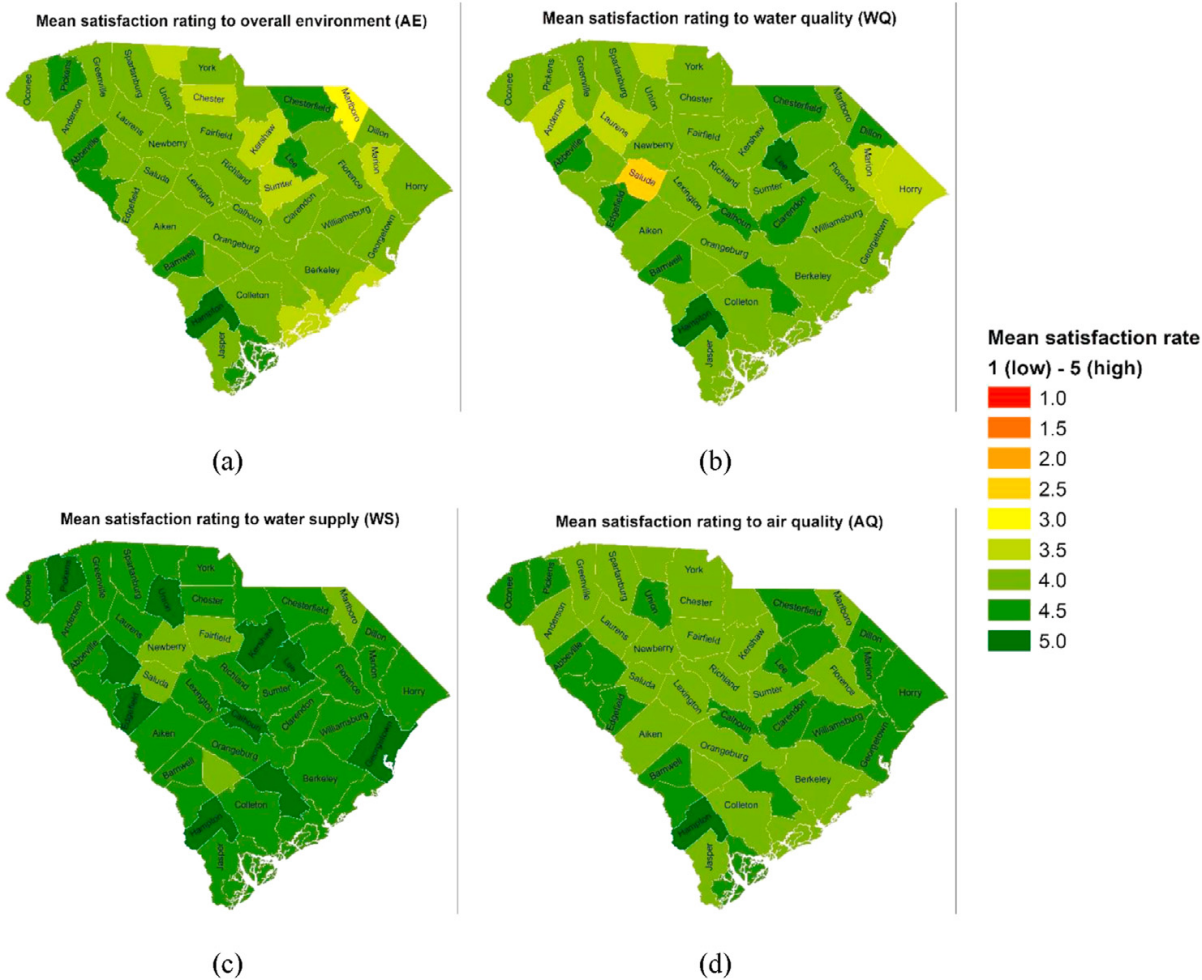
Geographic distribution of satisfaction rating per county by environmental characteristics. a shows the mean satisfaction rating on the overall state of the environment; b shows the mean satisfaction rating on water quality; c shows the mean satisfaction rating on water supply; d shows the mean satisfaction rating on air quality.

Furthermore, the mean satisfaction rating by county was mapped in Figure 2, where the maps visualized the residents' satisfaction rating for each of the environmental characteristics. Dark green color indicates a higher satisfaction rating, while lighter green color indicates a lower satisfaction rating. Colors ranging from yellow, orange, and red indicate a range from neither satisfied nor dissatisfied, moderately dissatisfied, and extremely dissatisfied, respectively.

Figure 2 shows that two counties, Marlboro and Saluda, rated relatively low in the overall quality of the environment and the water quality. The dissatisfied rating for water quality in Saluda county could be due to a report where the maximum contaminant level of total trihalomethanes (TTHM) exceeded the threshold (Saluda County Water and Sewer Authority, 2019). Because the survey was conducted near the period when this was reported to the public, this incident could have affected the residents' perception.

Although satisfaction ratings are not cardinal values, the result indicates that SC residents are satisfied with the amount of water they can access and the quality of air within their area. On the other hand, while the perceived satisfaction with water quality and overall quality of the environment is lower than the other two characteristics, this could serve as a baseline on the residents' perception. Therefore, future interventions can use these baseline satisfaction ratings to validate the program's effectiveness or further investigate possible issues and opportunities that could affect the residents' satisfaction.

### 3.3 Assessing the residents' preference towards priority ecosystem service and ecosystem

#### 3.3.1 Analysis of residents' preference to priority ecosystem service for a conservation program targeting

**Figure 3:**
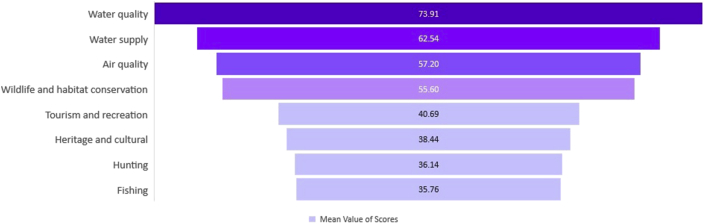
The rank of Ecosystem Service preference using “mean value of scores” from Garrett ranking analysis.

Results of the Garrett ranking analysis (Appendix 6) are shown in Figure 3. Using the mean value of ranking scores, the results show that residents prioritize the conservation of water-related ecosystem services, particular water quality. On the other hand, the least priorities are hunting and fishing. The results of the ecosystem service ranking indicate that stakeholders recognize the need for improving the water quality in the state.

Another notable result from the rank analysis is the rank between “air quality” and “wildlife and habitat conservation.” Although, as reflected in the satisfaction rating, respondents seem to be pleased with the state of air quality, it is also almost tied up with wildlife and habitat conservation. This goes to show that SC residents also place a high priority on the conservation of wildlife. One possible reason for this observation could be a socio-cultural attribution of wildlife-associated recreational activities in SC. This can also be commonly observed particularly in the southern region of the United States. Since wildlife-associated recreation generates economic benefits for the state of SC (Willis and Straka, 2016), this plays an important influence on the prioritization preference of the residents.

Since the rank analysis identified water quality as the priority ecosystem service, we analyzed the possible factors which lead to this preference. Using the Multi-Logit model, we compared respondents' likelihood to choose water quality over water supply and other “non-water related” ES.

The model evaluated the respondents' likely choice of priority ecosystem service between the baseline priority ES - water quality - and two other alternatives: water supply and other ES. Table 5 shows the result of the multinomial logit regression displaying the factors affecting the respondent's preference and the probability of the respondent to choose between the baseline as compared to the alternative.

**Table 5:** Multi-Logit regression^1^ of resident's priority ecosystem service.

Predictor	vs. Water Supply		vs. other ES	
	Coef (SE)	Relative risk ratio	Coef (SE)	Relative risk ratio
Intercept	-1.882∗∗∗ (0.40)	0.15	-0.502ˆ (0.28)	0.61
Satisfaction rating to water quality (satisfied)	0.620∗ (0.24)	1.86	0.326∗ (0.16)	1.38
Satisfaction rating to water supply (satisfied)	-0.908∗∗∗ (0.27)	0.40	-0.034 (0.20)	0.97
Satisfaction rating to overall environmental quality (satisfied)	0.002 (0.22)	1.00	-0.117 (0.15)	0.89
Age	0.003 (0.01)	1.00	-0.014∗∗∗ (0.00)	0.99
Income $50,000 - $99,999	0.182 (0.20)	1.20	-0.206ˆ (0.14)	0.81
Income more than $100,000	-0.082 (0.26)	0.92	-1.013∗∗∗ (0.22)	0.36
Distance to an impaired stream	-0.003 (0.07)	1.00	0.170∗∗ (0.05)	1.18
Distance to a good water body	0.053 (0.09)	1.05	0.015 (0.06)	1.02
Residents from the midland region	-0.086 (0.22)	0.92	0.107 (0.15)	1.11
Residents from the upstate region	0.222 (0.23)	1.25	0.319ˆ (0.16)	1.38

Likelihood ratio test: chisq = 77.603 (p.value = 9.9815e-09).

McFadden Rˆ2: 0.03.

∗∗∗ pval <0.001.

∗∗ pval <0.01.

∗ pval <0.05.

ˆ pval <0.10.

1 The Multi-Logit regression coefficient shows the log-odds ratio while the relative risk ratio is the probability associated to the likelihood of the respondent's choice considering the independent variable as the respondent's characteristic. A value of 1 means that there is no change, hence the respondents are indifferent between the alternative and the baseline. A RR ratio greater than 1 represents that respondents have higher likelihood to choose the alternative compared to the baseline. The Multi-Logit regression model was ran in the R Studio software using the “mlogit” package.

##### 3.3.1.1 Likelihood of choosing water quality vs. water supply as the preferred priority ecosystem service

Results in Table 5 show that the satisfaction ratings towards water quality and water supply are likely to affect residents' preference. Notably, residents who are satisfied with their current water quality are 86% more likely to prioritize water supply. On the other hand, residents who are satisfied with their water supply are 60% more likely to prioritize water quality as the target for conservation programs. This shows that residents' prioritization towards water quality regulation as ES, although more preferred by respondents, does not mean that water supply should not be prioritized at all. Water quality regulation and water supply provision, although different ecosystem services, are usually dealt with and managed together (Vigerstol and Aukema, 2011; Cosgrove and Loucks, 2015; Bai et al., 2019).

##### 3.3.1.2 Likelihood of choosing water quality vs. other non-water related ES as preferred priority ecosystem service

Comparing the resident's preference between water quality and other ES showed more variables affecting their choices, namely: satisfaction rating to water quality, socio-economic factors such as age and income, proximity to the nearest impaired stream, and if residents' household is located in the upstate region.

Similar to the water supply, respondents that are satisfied with the quality of water are 38% more likely to prioritize other ES. This could imply that only when respondents are satisfied with water quality will they be more likely to prioritize other ES. This follows the results of previous studies showing that residents prioritize the improvement of water quality (Brox et al., 1996; Calderon et al., 2012; Doherty et al., 2014; Castro et al., 2016; Aguilar et al., 2018; Khan et al., 2019; Khan and Zhao, 2019).

Meanwhile, socio-economic covariates suggest that older respondents have a higher likelihood to prioritize water quality. Particularly, as respondents increase their age by a year, the likelihood that they will choose water quality as compared to other ES increases by 1%. This could possibly be associated with house ownership. Older respondents are typically homeowners (70% of the respondents), where access to adequate water quality is an essential component in owning a house in a specific area.

Furthermore, in terms of income levels, results show that households with higher income levels are more likely to prioritize water quality than other ES. Particularly, households with an annual income of $50,000 to $99,999 are 19% more likely to prioritize water quality as compared to households with income lower than $50,000. Moreover, households with an annual income of more than $100,000 are 64% more likely to prioritize water quality as compared to households with income lower than $50,000. This could be associated with the cost of accessing a good quality of water. In recent years, households install filtration systems or simply buy bottled water to ensure high water quality for consumption (Quick, 2018). Households with income higher than the state's mean household income of $72,000 (SC Department of Employment and Workforce, 2018; United States Census Bureau, 2018) implies more capable of installing filtration systems while the other less expensive alternative is to buy bottled water. Therefore, an improvement in the water quality could decrease these costs for households. In any case, the income variable showed that water quality is more likely to be prioritized by residents as compared to other non-water related ecosystem services.

In terms of the proximity to monitored ecosystems, only the distance to impaired streams showed a statistically significant effect. In this case, a 1-kilometer increase in distance between the respondent's household from the nearest impaired stream implies that these respondents are 18% more likely to choose other ES to be prioritized rather than water quality. This could be because respondents living farther from an impaired stream do not see or are not aware of an impaired stream's negative impact. Hence, the likelihood of prioritizing water quality over other ES also decreases.

Residents living in the upstate region are 38% more likely to prioritize other ES compared to those in the low country or coastal areas. This could be attributed to the satisfaction rating of the upstate residents to water quality. Overall, 87% of the respondents from the upstate gave a satisfactory rating to the water quality, while 85% for the low country. Since more residents in the upstate are satisfied with the water quality, this could be the reason why they are more likely to choose other ES as compared to low country/coastal residents.

Overall, the result for the intercepts in both comparisons showed to be statistically significant favoring water quality. This indicates that suppose all other factors are constant, respondents are more likely to prioritize water quality than water supply or other ES.

#### 3.3.2 Analysis of residents' preference to priority ecosystem for conservation program intervention

**Figure 4:**
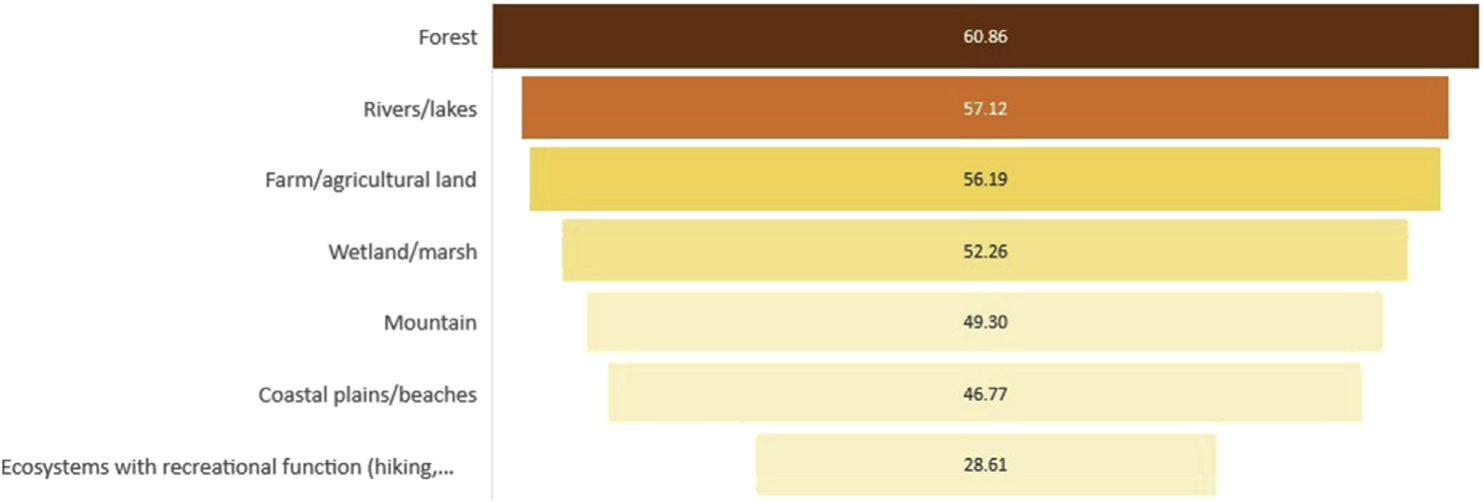
The rank of Ecosystem preference using “mean value of scores” from Garrett ranking analysis.

As with the ecosystem service preference analysis, we analyzed respondents' preference towards prioritization of the ecosystem. Similarly, understanding these preferences towards priority ecosystems can narrow the appropriate conservation program recommendation for targeting the preferred ecosystem service. As with the ES ranking analysis, the same methodology was used for the priority ecosystem (Appendix 8), and the result of the rank analysis is shown in Figure 4.

The hierarchy showed that the forest ecosystem is the main priority for respondents. The analysis also indicated a very small difference in preferences between rivers/lakes and farm/agricultural land. Despite the tight rank score difference of the next best alternatives, it is still clear that the top priority ecosystem is directly related to the improvement of water-related ecosystem services. This result was consistent with the stakeholders' preference towards the priority ecosystem service discussed in the previous section.

The results of the Multi-Logit regression for the priority ecosystem preference is shown in Table 6. We used “forest” as the baseline while the river ecosystem, agriculture ecosystem, and other ecosystems were the alternatives.

**Table 6:** Multi-Logit regression of resident's priority ecosystem.

Predictor	vs. River		vs. Agri		vs. others	
	Coef (SE)	Relative risk ratio	Coef (SE)	Relative risk ratio	Coef (SE)	Relative risk ratio
Intercept	-1.585∗∗∗ (0.37)	0.20	-0.867∗ (0.34)	0.42	-0.410 (0.32)	0.66
Satisfaction rating to water quality (satisfied)	-0.060 (0.20)	0.94	-0.200 (0.18)	0.82	0.120 (0.19)	1.13
Satisfaction rating to water supply (satisfied)	0.352 (0.25)	1.42	0.737∗∗ (0.23)	2.09	0.287 (0.23)	1.33
Satisfaction rating to overall environmental quality (satisfied)	0.083 (0.20)	1.09	-0.175 (0.18)	0.84	0.055 (0.18)	1.06
Age	0.017∗∗∗ (0.00)	1.02	0.011∗ (0.00)	1.01	0.015∗∗ (0.00)	1.01
Income $50,000 - $99,999	-0.255 (0.17)	1.29	0.079 (0.16)	1.08	0.017 (0.16)	1.02
Income more than $100,000	-0.262 (0.24)	1.30	0.046 (0.23)	1.05	0.378 (0.22)	1.46
Distance to an impaired stream	0.038 (0.07)	1.04	0.059 (0.06)	1.06	0.047 (0.06)	1.05
Distance to a good water body	0.023 (0.08)	1.02	0.126ˆ (0.07)	1.13	-0.026 (0.07)	0.97
Residents from the midland region	0.148 (0.20)	1.16	-0.473∗∗ (0.18)	0.62	-0.816∗∗∗ (0.18)	0.44
Residents from the upstate region	0.145 (0.21)	1.16	-0.221 (0.19)	0.80	-0.724∗∗∗ (0.19)	0.49

Likelihood ratio test: chisq = 114.78 (p.value = 5.69e-11).

McFadden Rˆ2: 0.03.

∗∗∗ pval <0.001.

∗∗ pval <0.01.

∗ pval <0.05.

ˆ pval <0.10.

Among all the covariates in the results shown in Table 6, only “age” showed a statistically significant effect across all ecosystem comparison, indicating that younger respondents are more likely to prioritize the forest ecosystem for conservation. This could be related to SC residents' high involvement in outdoor activities, particularly for young and middle-aged residents. Outdoor activities, including hunting, recreational fishing, and water recreation activities, substantially contribute to the state's economy (Willis and Straka, 2016). It was claimed that, on average, SC residents participate in fishing and hunting more than the average American (Outdoor Industry Association, 2019). Conservation of the forest ecosystem maintains the trails, the quality of rivers and streams, and wildlife habitat, making it conducive to outdoor activities.

##### 3.3.2.1 Likelihood of choosing forest ecosystem vs. river ecosystem as the preferred priority ecosystem

Results presented in Table 6 show that apart from the respondent's age and the intercept, non among the other variables that we have investigated showed statistically significant evidence that residents prioritize the forest ecosystem compared to the river ecosystem. However, the intercept indicates that, if all variables are held constant, residents are 80% more likely to prioritize forest ecosystems for conservation programs as compared to the river ecosystem.

##### 3.3.2.2 Likelihood of choosing forest ecosystem vs. agricultural ecosystem as the preferred priority ecosystem

Since one of the major beneficiaries of ecosystem services is agriculture, we also compared the respondents' prioritization between forest ecosystems and agroecosystems. Agriculture is also the third-ranked priority ecosystem in the earlier rank analysis in Figure 4.

Results in Table 6 show that respondents who are primarily satisfied with the current water supply are 109% more likely to choose the agriculture ecosystem than the forest ecosystem to be prioritized. Residents' perception of the abundance of water in SC could be why they choose agriculture activities. Residents may believe there is enough water for crop production in the state.

In terms of the distance to monitored ecosystems, results in Table 6 showed that as the distance of the center of the household's zip code goes farther from a good water body, the respondent is more likely to choose the agroecosystem the priority ecosystem to be conserved by 13%. This result could be attributed to SC residents preferring agricultural land to forest land. Mainly if they are located in an agriculturally dominated area or agriculture is a significant income source in their household, such as in the midland region of SC. South Carolina has historically been dependent on natural resources, including agriculture, for its economic growth (Willis and Straka, 2016). However, while urbanization and industrial areas continue to develop, the agriculture industry continues to decline. In fact, the GDP contribution from farms in 1997 only amounts to 0.76% and continues to decline, amounting to 0.30% in 2017 (US Bureau of Economic Analysis). However, around 14% of the land is still classified as agricultural (US Geological Survey (USGS) Gap Analysis Project (GAP), 2012); hence, this shows that a substantial amount of households are heavily dependent on agriculture. Respondents under these conditions tend to prioritize the improvement of the agroecosystem rather than the forest ecosystem.

On the other hand, respondents residing in the midland region are 38% more likely to prioritize the forest ecosystem as compared to those who reside in the low country. The midland region showed a low satisfaction rating for water quality relative to the other regions (Figure 3b). Therefore, since enhanced forest management is attributed to an improvement in water quality (Aguilar et al., 2018), the urgency from the residents' satisfaction rating likely contributes to their preference for prioritizing the forest ecosystem for conservation.

Lastly, the intercept also shows that holding all other factors as constant, residents are more likely to prioritize forest ecosystems by 58% as compared to the agriculture ecosystem.

##### 3.3.2.3 Likelihood of choosing forest ecosystem vs. “other ecosystems” as preferred priority ecosystem

In comparison to “other ecosystems,” results in Table 6 revealed that the residents' geographic region indicates statistically significant evidence that it affects the respondents' preference.

Residents from both midland and the upstate are 51%–56% more likely to choose the forest ecosystem for conservation as compared to residents from the low country. While the midland residents' preference could be attributed to their satisfaction rating to water quality, the upstate's preference could be attributed to the land cover. Since the topography of the upstate is hilly and mountainous, the majority of the land cover classified as forested area are in this region (US Geological Survey (USGS) Gap Analysis Project (GAP), 2012). Therefore, upstate residents have better familiarity with the ecosystem and ecosystem services of the forest which benefits them. Hence, they choose to prioritize the forest ecosystem.

Overall, the results reinforce the rank analysis in which the residents of SC prefer to prioritize forest ecosystems for conservation compared to other ecosystems. Essentially, respondents to our survey revere to prioritize the forest ecosystem because they are aware that it is the main source of the primary ecosystem services in SC which directly benefits them.

## 4 Summary and conclusion

This study examined the priorities for conservation of ecosystems and ecosystem services for residents in South Carolina, USA. Since residents are typically the final recipients of ecosystem services, identification of priority ecosystem and ecosystem services from the public's outlook could help in the strategic implementation of conservation programs. Nevertheless, to the best of our knowledge, no study has previously investigated SC residents' preference regarding ES.

Results showed that SC residents are likely to prioritize water-related ecosystem services, particularly the improvement to water quality regulation, while their least priorities are fishing and hunting. Although South Carolina residents are actively involved in fishing, hunting, and outdoor activities, water quality improvement still poses to be their top priority since water quality improvement also benefits other ecosystem services, including fishing and other outdoor activities. Similarly, this was also reflected in the respondents' mean satisfaction rating through their general impression of the current state of the environment in SC. The satisfaction rating to the water quality and the impression of the overall quality of the environment scored lowest as compared to water supply and air quality. While further investigation is still needed to determine the specific reason why this is the case, the results of this study could be used as a baseline for monitoring these characteristics.

Furthermore, these results are consistent with the residents' preference to prioritize water quality regulation as the primary ecosystem service for conservation program targeting. However, their preferences towards water quality improvement do not discredit the importance of maintaining the continuous water supply provision, as was evident in the maximum likelihood analysis. Most covariates did not yield statistically significant evidence when comparing water quality and water supply as the priority ecosystem service. Therefore, as state resource managers continually update their water plans, including monitoring systems for water quality improvement would ensure that they address the challenge of meeting the water demand while also meeting the public's satisfaction standards for water quality.

In terms of ecosystem preference, respondents to our survey indicated that the forest ecosystem is the priority ecosystem to be conserved. The reasons for this preference also align with their ecosystem service preference. Respondents are aware of the forest ecosystem's direct linkage to water-related ecosystem services; therefore, they opt to choose to conserve the ecosystem that also enhances their primary priority ecosystem service. Thus, in planning for conservation interventions, prioritizing the conservation programs for the forested land would reap more support and possible participation from the public.

The prioritization ranking of SC residents also revealed their preferences towards the primary ecosystem and ecosystem service for conservation. Apart from the satisfaction ratings, socio-economic factors such as age and income also showed statistical evidence that affects the respondents' prioritization. Also, proximities to monitored ecosystems revealed to have a significant contribution in evaluating respondents' preferences. The regression result using the proximity of monitored ecosystems also showed that the quality of these ecosystems affects the residents' perception and prioritization criteria. For instance, residents who live farther from an impaired stream do not see the urgency of an improved water body hence will prioritize other ecosystem services more than water quality regulation.

On the other hand, those who live in agriculture-dependent areas and near a good water body will prioritize agriculture ecosystem rather than forest ecosystems because of the availability of water that could be used for irrigation purposes. Furthermore, the geographic region of the respondents showed a statistically significant contribution affecting their preference. Since geographic regions have different landscape characteristics such as topography and land cover, this could also affect the residents' preference for conservation. Therefore, the results showed that perception and impressions of nearby ecosystems and their geographic location affect their preferences and prioritization. This analysis could be important in targeting the stakeholders that could be involved in supporting the conservation programs. For instance, since younger residents and residents with higher income are keen on forest conservation, designing sustainable financing mechanisms or user-fee mechanisms could be tailor-fitted to this group. Knowing the residents' priority ecosystem and ecosystem service for conservation is an essential initial step for conducting a WTP study for conservation planning activities and as an economic basis for developing sustainable financing mechanisms that will support conservation programs.

The use of SC residents' perception, including satisfaction rating, to measure the public's general impression towards the environment served as a feedback mechanism. Ensuring that the public's satisfaction standards are met translates into public support, hence could increase the potential funding support for conservation programs. Understanding the results from their perception can draw up insights for crafting strategic implementation of conservation programs and further conservation studies. For instance, in 2013, in addressing the water supply problem, the state tapped the residents' ability to promote the efficient use of water through the “WaterSense” program (US Environmental Protection Agency, 2013). The program encouraged residents to install WaterSense labeled products to ensure that their households are using water-saving technologies. The program was advertised and popularized by the “Every Drop Counts” campaign of the state, which led to a savings of 677 million gallons of water annually (US Environmental Protection Agency, 2013). This program proved that residents' participation and preferences could improve the implementation of conservation programs. Therefore, the results of this study could provide important information on implementing conservation programs, particularly in focusing on water quality and the forest ecosystem.

As the state water plans are continuously being updated by the state agencies and the South Carolina Water Resources Center (SCWRC) to ensure that there is enough supply of water for everyone, this study showed that there should also be a focus on the water quality regulation and ecosystem conservation, particularly towards the forested land. Picking up from the results of this study, further research endeavors focusing on water-related ecosystem services in SC could provide better assessment and information about their conservation program priorities. Furthermore, knowing the stakeholders' priority ecosystem and ecosystem services can be used for designing specific valuation studies.

Since the study was focused on residents as the main stakeholder, further research will be to look at other stakeholders' perspectives such as farmers, landowners, tourists, and businesses, which could provide more insights on the feasibility of implementing conservation programs. Also, since the study was conducted on an online platform, the results of this study are limited to inference regarding only residents with access to the internet. Although most residents across the state have internet access, a substantial number of residents are still without online access. Therefore, it is worth pursuing to examine the preferences of non-internet users on the matter. Moreover, while the scale of the study focuses only on SC residents, it will be interesting for future research to compare the residents' preferences across different states or regions. This comparison could provide a more comprehensive assessment of the decision-making factors that affect an individual's preference to prioritize an ecosystem or ecosystem service for conservation. Finally, in pursuing sustainability as defined in the World Commission on Environment and Development (WCED), future research and ES approaches should include a more diverse notion of social-ecological systems by making it centered towards the stakeholder while not compromising the ecosystem integrity. Therefore, future managers can draw insights from the results of this study to craft strategic implementation of conservation programs by incorporating the residents' preference.

## Declarations

### Author contribution statement

J. Carl Ureta: Conceived and designed the experiments; Performed the experiments; Analyzed and interpreted the data; Contributed reagents, materials, analysis tools or data; Wrote the paper.

Michael Vassalos, Marzieh Motallebi: Conceived and designed the experiments; Analyzed and interpreted the data; Contributed reagents, materials, analysis tools or data; Wrote the paper.

Robert Baldwin: Conceived and designed the experiments; Contributed reagents, materials, analysis tools or data.

Joan Ureta: Analyzed and interpreted the data; Contributed reagents, materials, analysis tools or data; Wrote the paper.

### Funding statement

This work was supported by the 10.13039/100005825National Institute of Food and Agriculture/USDA (Award #: 2018-67020-27854) and the South Carolina 10.13039/100009171Natural Resources Conservation Service (Award #: NR184639XXXXG002).

### Data availability statement

Data will be made available on request.

### Declaration of interests statement

The authors declare no conflict of interest.

### Additional information

No additional information is available for this paper.

## Footnotes

1 Stakeholders are any group or individual that can affect or be affected by the ecosystem and ecosystem services (Hein et al., 2006).

2 The vast majority of the forest land (86%) is privately owned (Butler and Wear 2013; South Carolina Forestry Commission 2015).
