# Supplementary material for: Using stakeholders' preference for ecosystems and ecosystem services as an economic basis underlying strategic conservation planning
Source: Heliyon. 2020 Dec 23;6(12):e05827. doi: 10.1016/j.heliyon.2020.e05827 (PMC7773879; doi:10.1016/j.heliyon.2020.e05827)
Supplement: Appendix [file mmc1.docx]

## Appendices

### Appendix 1 Garrett Ranking Conversion Table.


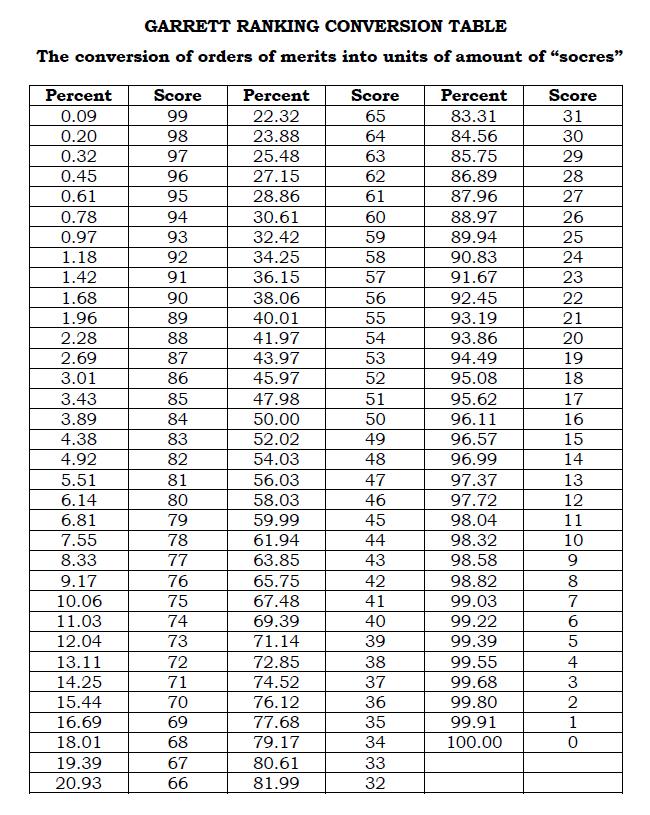


Adopted from: (Sedaghat 2011; Dhanavandan 2016; Arunkaumar et al. 2018)

### Appendix 2 Satisfaction rating summary towards current state of water quality.

| **County** | **% of samples within the county that rated 1 for water quality** | **% of samples within the county that rated 2 for water quality** | **% of samples within the county that rated 3 for water quality** | **% of samples within the county that rated 4 for water quality** | **% of samples within the county that rated 5 for water quality** | **Mean of satisfaction score for "water quality" with 1 being the lowest and 5 being the highest** |
| --- | --- | --- | --- | --- | --- | --- |
| **Abbeville** | 0.0 | 0.1 | 0.2 | 0.2 | 0.5 | 4.1 |
| **Aiken** | 0.0 | 0.2 | 0.1 | 0.4 | 0.3 | 3.8 |
| **Anderson** | 0.0 | 0.2 | 0.2 | 0.3 | 0.2 | 3.5 |
| **Bamberg** | 0.1 | 0.0 | 0.1 | 0.4 | 0.3 | 3.7 |
| **Barnwell** | 0.1 | 0.0 | 0.1 | 0.3 | 0.6 | 4.3 |
| **Beaufort** | 0.0 | 0.1 | 0.1 | 0.4 | 0.3 | 3.9 |
| **Berkeley** | 0.0 | 0.1 | 0.2 | 0.4 | 0.4 | 3.9 |
| **Calhoun** | 0.0 | 0.0 | 0.2 | 0.2 | 0.6 | 4.4 |
| **Charleston** | 0.1 | 0.1 | 0.1 | 0.5 | 0.3 | 3.8 |
| **Cherokee** | 0.0 | 0.2 | 0.2 | 0.4 | 0.2 | 3.5 |
| **Chester** | 0.0 | 0.1 | 0.1 | 0.3 | 0.4 | 4.0 |
| **Chesterfield** | 0.0 | 0.0 | 0.2 | 0.3 | 0.5 | 4.2 |
| **Clarendon** | 0.1 | 0.0 | 0.1 | 0.3 | 0.5 | 4.2 |
| **Colleton** | 0.1 | 0.2 | 0.0 | 0.5 | 0.2 | 3.7 |
| **Darlington** | 0.0 | 0.0 | 0.2 | 0.5 | 0.3 | 3.9 |
| **Dillon** | 0.0 | 0.3 | 0.0 | 0.0 | 0.7 | 4.1 |
| **Dorchester** | 0.0 | 0.0 | 0.1 | 0.5 | 0.4 | 4.2 |
| **Edgefield** | 0.1 | 0.0 | 0.1 | 0.2 | 0.6 | 4.3 |
| **Fairfield** | 0.0 | 0.0 | 0.3 | 0.3 | 0.3 | 4.0 |
| **Florence** | 0.0 | 0.1 | 0.2 | 0.4 | 0.2 | 3.8 |
| **Georgetown** | 0.1 | 0.1 | 0.1 | 0.2 | 0.4 | 3.7 |
| **Greenville** | 0.0 | 0.1 | 0.1 | 0.4 | 0.3 | 3.9 |
| **Greenwood** | 0.0 | 0.1 | 0.1 | 0.5 | 0.3 | 3.9 |
| **Hampton** | 0.0 | 0.0 | 0.0 | 0.0 | 1.0 | 5.0 |
| **Horry** | 0.1 | 0.2 | 0.2 | 0.4 | 0.2 | 3.5 |
| **Jasper** | 0.0 | 0.0 | 0.0 | 1.0 | 0.0 | 4.0 |
| **Kershaw** | 0.0 | 0.2 | 0.1 | 0.5 | 0.2 | 3.7 |
| **Lancaster** | 0.1 | 0.1 | 0.1 | 0.4 | 0.3 | 3.8 |
| **Laurens** | 0.0 | 0.2 | 0.2 | 0.4 | 0.2 | 3.5 |
| **Lee** | 0.0 | 0.0 | 0.0 | 0.3 | 0.7 | 4.7 |
| **Lexington** | 0.1 | 0.1 | 0.2 | 0.4 | 0.3 | 3.7 |
| **Marion** | 0.1 | 0.1 | 0.1 | 0.4 | 0.1 | 3.3 |
| **Marlboro** | 0.1 | 0.0 | 0.1 | 0.3 | 0.4 | 3.9 |
| **McCormick** | 0.1 | 0.0 | 0.0 | 0.7 | 0.2 | 3.9 |
| **Newberry** | 0.1 | 0.1 | 0.1 | 0.4 | 0.4 | 4.0 |
| **Oconee** | 0.0 | 0.2 | 0.1 | 0.5 | 0.3 | 3.9 |
| **Orangeburg** | 0.1 | 0.2 | 0.0 | 0.4 | 0.3 | 3.6 |
| **Pickens** | 0.0 | 0.1 | 0.2 | 0.4 | 0.3 | 3.9 |
| **Richland** | 0.0 | 0.1 | 0.1 | 0.5 | 0.3 | 4.0 |
| **Saluda** | 0.3 | 0.3 | 0.0 | 0.3 | 0.0 | 2.3 |
| **Spartanburg** | 0.0 | 0.1 | 0.2 | 0.4 | 0.3 | 3.8 |
| **Sumter** | 0.1 | 0.1 | 0.1 | 0.6 | 0.2 | 3.8 |
| **Union** | 0.0 | 0.0 | 0.0 | 1.0 | 0.0 | 4.0 |
| **Williamsburg** | 0.0 | 0.1 | 0.2 | 0.5 | 0.2 | 3.9 |
| **York** | 0.0 | 0.1 | 0.2 | 0.4 | 0.3 | 3.8 |
| **MEDIAN** |  |  |  |  |  | 3.9 |

### Appendix 3 Satisfaction rating summary towards current state of water supply.

| **County** | **% of samples within the county that rated 1 for water supply** | **% of samples within the county that rated 2 for water supply** | **% of samples within the county that rated 3 for water supply** | **% of samples within the county that rated 4 for water supply** | **% of samples within the county that rated 5 for water supply** | **Mean of satisfaction score for "water supply" with 1 being the lowest and 5 being the highest** |
| --- | --- | --- | --- | --- | --- | --- |
| **Abbeville** | 0.0 | 0.1 | 0.1 | 0.3 | 0.5 | 4.2 |
| **Aiken** | 0.0 | 0.0 | 0.1 | 0.3 | 0.6 | 4.4 |
| **Anderson** | 0.0 | 0.0 | 0.1 | 0.4 | 0.5 | 4.3 |
| **Bamberg** | 0.1 | 0.0 | 0.1 | 0.3 | 0.4 | 3.9 |
| **Barnwell** | 0.0 | 0.1 | 0.2 | 0.3 | 0.5 | 4.2 |
| **Beaufort** | 0.0 | 0.0 | 0.1 | 0.2 | 0.7 | 4.5 |
| **Berkeley** | 0.0 | 0.0 | 0.0 | 0.3 | 0.6 | 4.4 |
| **Calhoun** | 0.0 | 0.0 | 0.0 | 0.4 | 0.6 | 4.6 |
| **Charleston** | 0.0 | 0.0 | 0.1 | 0.2 | 0.6 | 4.3 |
| **Cherokee** | 0.0 | 0.1 | 0.1 | 0.2 | 0.6 | 4.3 |
| **Chester** | 0.0 | 0.0 | 0.1 | 0.6 | 0.4 | 4.3 |
| **Chesterfield** | 0.0 | 0.0 | 0.1 | 0.2 | 0.7 | 4.5 |
| **Clarendon** | 0.0 | 0.0 | 0.1 | 0.4 | 0.5 | 4.4 |
| **Colleton** | 0.1 | 0.0 | 0.1 | 0.2 | 0.6 | 4.3 |
| **Darlington** | 0.0 | 0.0 | 0.1 | 0.4 | 0.4 | 4.2 |
| **Dillon** | 0.0 | 0.0 | 0.3 | 0.3 | 0.4 | 4.1 |
| **Dorchester** | 0.0 | 0.0 | 0.1 | 0.2 | 0.7 | 4.6 |
| **Edgefield** | 0.0 | 0.1 | 0.0 | 0.1 | 0.8 | 4.6 |
| **Fairfield** | 0.0 | 0.3 | 0.0 | 0.3 | 0.3 | 3.7 |
| **Florence** | 0.0 | 0.1 | 0.1 | 0.4 | 0.5 | 4.2 |
| **Georgetown** | 0.0 | 0.0 | 0.1 | 0.2 | 0.7 | 4.6 |
| **Greenville** | 0.0 | 0.0 | 0.1 | 0.3 | 0.5 | 4.3 |
| **Greenwood** | 0.0 | 0.0 | 0.1 | 0.3 | 0.6 | 4.6 |
| **Hampton** | 0.0 | 0.0 | 0.0 | 0.0 | 1.0 | 5.0 |
| **Horry** | 0.0 | 0.0 | 0.1 | 0.2 | 0.6 | 4.3 |
| **Jasper** | 0.0 | 0.0 | 0.0 | 0.6 | 0.4 | 4.4 |
| **Kershaw** | 0.0 | 0.0 | 0.0 | 0.3 | 0.7 | 4.6 |
| **Lancaster** | 0.1 | 0.0 | 0.1 | 0.2 | 0.6 | 4.2 |
| **Laurens** | 0.0 | 0.0 | 0.2 | 0.2 | 0.6 | 4.2 |
| **Lee** | 0.0 | 0.0 | 0.0 | 0.3 | 0.7 | 4.7 |
| **Lexington** | 0.0 | 0.0 | 0.1 | 0.4 | 0.5 | 4.2 |
| **Marion** | 0.0 | 0.0 | 0.1 | 0.4 | 0.4 | 4.3 |
| **Marlboro** | 0.1 | 0.0 | 0.1 | 0.4 | 0.3 | 3.7 |
| **McCormick** | 0.0 | 0.0 | 0.0 | 0.5 | 0.5 | 4.5 |
| **Newberry** | 0.1 | 0.1 | 0.3 | 0.2 | 0.4 | 3.8 |
| **Oconee** | 0.0 | 0.1 | 0.0 | 0.2 | 0.7 | 4.5 |
| **Orangeburg** | 0.0 | 0.1 | 0.1 | 0.3 | 0.5 | 4.2 |
| **Pickens** | 0.0 | 0.0 | 0.1 | 0.2 | 0.7 | 4.7 |
| **Richland** | 0.0 | 0.0 | 0.2 | 0.2 | 0.6 | 4.4 |
| **Saluda** | 0.0 | 0.3 | 0.0 | 0.3 | 0.3 | 3.7 |
| **Spartanburg** | 0.0 | 0.0 | 0.1 | 0.2 | 0.6 | 4.4 |
| **Sumter** | 0.0 | 0.0 | 0.1 | 0.5 | 0.4 | 4.2 |
| **Union** | 0.0 | 0.0 | 0.0 | 0.2 | 0.8 | 4.8 |
| **Williamsburg** | 0.0 | 0.1 | 0.2 | 0.3 | 0.4 | 4.1 |
| **York** | 0.0 | 0.1 | 0.2 | 0.3 | 0.5 | 4.2 |
| **MEDIAN** |  |  |  |  |  | 4.3 |

### Appendix 4 Satisfaction rating summary towards current state of air quality.

| **County** | **% of samples within the county that rated 1 for air quality** | **% of samples within the county that rated 2 for air quality** | **% of samples within the county that rated 3 for air quality** | **% of samples within the county that rated 4 for air quality** | **% of samples within the county that rated 5 for air quality** | **Mean of satisfaction score for "air quality" with 1 being the lowest and 5 being the highest** |
| --- | --- | --- | --- | --- | --- | --- |
| **Abbeville** | 0.0 | 0.1 | 0.1 | 0.1 | 0.7 | 4.4 |
| **Aiken** | 0.0 | 0.1 | 0.1 | 0.3 | 0.4 | 4.0 |
| **Anderson** | 0.0 | 0.1 | 0.1 | 0.5 | 0.3 | 3.9 |
| **Bamberg** | 0.1 | 0.0 | 0.3 | 0.1 | 0.4 | 3.7 |
| **Barnwell** | 0.1 | 0.1 | 0.1 | 0.2 | 0.6 | 4.1 |
| **Beaufort** | 0.0 | 0.0 | 0.1 | 0.4 | 0.5 | 4.3 |
| **Berkeley** | 0.1 | 0.1 | 0.1 | 0.4 | 0.3 | 3.8 |
| **Calhoun** | 0.0 | 0.0 | 0.0 | 0.6 | 0.4 | 4.4 |
| **Charleston** | 0.0 | 0.1 | 0.2 | 0.4 | 0.2 | 3.8 |
| **Cherokee** | 0.0 | 0.1 | 0.3 | 0.4 | 0.2 | 3.7 |
| **Chester** | 0.0 | 0.3 | 0.1 | 0.1 | 0.4 | 3.7 |
| **Chesterfield** | 0.0 | 0.1 | 0.1 | 0.3 | 0.5 | 4.3 |
| **Clarendon** | 0.0 | 0.1 | 0.1 | 0.3 | 0.5 | 4.3 |
| **Colleton** | 0.1 | 0.1 | 0.2 | 0.2 | 0.5 | 4.0 |
| **Darlington** | 0.0 | 0.0 | 0.2 | 0.5 | 0.3 | 4.2 |
| **Dillon** | 0.0 | 0.1 | 0.0 | 0.3 | 0.6 | 4.3 |
| **Dorchester** | 0.0 | 0.1 | 0.1 | 0.5 | 0.4 | 4.2 |
| **Edgefield** | 0.0 | 0.0 | 0.1 | 0.5 | 0.5 | 4.4 |
| **Fairfield** | 0.0 | 0.0 | 0.3 | 0.3 | 0.3 | 4.0 |
| **Florence** | 0.0 | 0.1 | 0.2 | 0.4 | 0.4 | 4.0 |
| **Georgetown** | 0.0 | 0.0 | 0.1 | 0.5 | 0.3 | 4.1 |
| **Greenville** | 0.0 | 0.1 | 0.2 | 0.4 | 0.3 | 3.9 |
| **Greenwood** | 0.0 | 0.0 | 0.1 | 0.5 | 0.4 | 4.3 |
| **Hampton** | 0.0 | 0.0 | 0.0 | 0.0 | 1.0 | 5.0 |
| **Horry** | 0.0 | 0.0 | 0.1 | 0.5 | 0.4 | 4.1 |
| **Jasper** | 0.0 | 0.0 | 0.2 | 0.8 | 0.0 | 3.8 |
| **Kershaw** | 0.0 | 0.1 | 0.1 | 0.5 | 0.3 | 4.0 |
| **Lancaster** | 0.1 | 0.1 | 0.1 | 0.3 | 0.4 | 4.0 |
| **Laurens** | 0.1 | 0.0 | 0.2 | 0.3 | 0.3 | 3.8 |
| **Lee** | 0.0 | 0.0 | 0.0 | 0.7 | 0.3 | 4.3 |
| **Lexington** | 0.0 | 0.0 | 0.2 | 0.5 | 0.3 | 3.9 |
| **Marion** | 0.0 | 0.0 | 0.3 | 0.1 | 0.6 | 4.3 |
| **Marlboro** | 0.1 | 0.0 | 0.0 | 0.6 | 0.3 | 3.9 |
| **McCormick** | 0.0 | 0.0 | 0.1 | 0.3 | 0.6 | 4.5 |
| **Newberry** | 0.0 | 0.1 | 0.0 | 0.6 | 0.3 | 4.0 |
| **Oconee** | 0.0 | 0.1 | 0.1 | 0.4 | 0.4 | 4.1 |
| **Orangeburg** | 0.0 | 0.1 | 0.2 | 0.4 | 0.3 | 3.9 |
| **Pickens** | 0.0 | 0.0 | 0.1 | 0.3 | 0.6 | 4.4 |
| **Richland** | 0.0 | 0.1 | 0.1 | 0.5 | 0.3 | 4.0 |
| **Saluda** | 0.0 | 0.3 | 0.0 | 0.3 | 0.3 | 3.7 |
| **Spartanburg** | 0.0 | 0.1 | 0.1 | 0.5 | 0.3 | 4.0 |
| **Sumter** | 0.0 | 0.1 | 0.2 | 0.4 | 0.3 | 3.8 |
| **Union** | 0.0 | 0.0 | 0.0 | 0.8 | 0.2 | 4.2 |
| **Williamsburg** | 0.0 | 0.1 | 0.1 | 0.4 | 0.4 | 4.1 |
| **York** | 0.0 | 0.1 | 0.2 | 0.4 | 0.3 | 3.9 |
| **MEDIAN** |  |  |  |  |  | 4.0 |

### Appendix 5 Satisfaction rating summary towards current state of the overall environment.

| **County** | **% of samples within the county that rated 1 for overall state of the environment** | **% of samples within the county that rated 2 for overall state of the environment** | **% of samples within the county that rated 3 for overall state of the environment** | **% of samples within the county that rated 4 for overall state of the environment** | **% of samples within the county that rated 5 for overall state of the environment** | **Mean of satisfaction score for "overall state of the environment" with 1 being the lowest and 5 being the highest** |
| --- | --- | --- | --- | --- | --- | --- |
| **Abbeville** | 0.1 | 0.0 | 0.1 | 0.3 | 0.5 | 4.1 |
| **Aiken** | 0.1 | 0.1 | 0.2 | 0.3 | 0.3 | 3.8 |
| **Anderson** | 0.0 | 0.1 | 0.2 | 0.5 | 0.1 | 3.6 |
| **Bamberg** | 0.1 | 0.0 | 0.3 | 0.1 | 0.4 | 3.7 |
| **Barnwell** | 0.0 | 0.1 | 0.1 | 0.3 | 0.5 | 4.3 |
| **Beaufort** | 0.0 | 0.0 | 0.1 | 0.6 | 0.3 | 4.1 |
| **Berkeley** | 0.0 | 0.1 | 0.3 | 0.3 | 0.2 | 3.6 |
| **Calhoun** | 0.0 | 0.0 | 0.2 | 0.6 | 0.2 | 4.0 |
| **Charleston** | 0.0 | 0.1 | 0.2 | 0.4 | 0.1 | 3.5 |
| **Cherokee** | 0.0 | 0.2 | 0.2 | 0.4 | 0.2 | 3.5 |
| **Chester** | 0.1 | 0.3 | 0.1 | 0.4 | 0.1 | 3.3 |
| **Chesterfield** | 0.0 | 0.1 | 0.1 | 0.4 | 0.5 | 4.1 |
| **Clarendon** | 0.0 | 0.3 | 0.1 | 0.5 | 0.2 | 3.6 |
| **Colleton** | 0.1 | 0.2 | 0.1 | 0.3 | 0.4 | 3.8 |
| **Darlington** | 0.0 | 0.2 | 0.2 | 0.4 | 0.3 | 3.7 |
| **Dillon** | 0.0 | 0.3 | 0.1 | 0.1 | 0.4 | 3.7 |
| **Dorchester** | 0.0 | 0.1 | 0.1 | 0.5 | 0.3 | 4.0 |
| **Edgefield** | 0.0 | 0.0 | 0.3 | 0.5 | 0.3 | 4.0 |
| **Fairfield** | 0.0 | 0.0 | 0.3 | 0.3 | 0.3 | 4.0 |
| **Florence** | 0.0 | 0.1 | 0.2 | 0.4 | 0.2 | 3.7 |
| **Georgetown** | 0.0 | 0.1 | 0.2 | 0.4 | 0.3 | 3.9 |
| **Greenville** | 0.0 | 0.1 | 0.2 | 0.5 | 0.2 | 3.8 |
| **Greenwood** | 0.0 | 0.1 | 0.2 | 0.5 | 0.3 | 4.0 |
| **Hampton** | 0.0 | 0.0 | 0.0 | 0.0 | 1.0 | 5.0 |
| **Horry** | 0.0 | 0.1 | 0.2 | 0.5 | 0.2 | 3.7 |
| **Jasper** | 0.0 | 0.0 | 0.4 | 0.6 | 0.0 | 3.6 |
| **Kershaw** | 0.0 | 0.3 | 0.0 | 0.5 | 0.2 | 3.5 |
| **Lancaster** | 0.0 | 0.1 | 0.1 | 0.5 | 0.3 | 3.9 |
| **Laurens** | 0.1 | 0.1 | 0.2 | 0.4 | 0.3 | 3.7 |
| **Lee** | 0.0 | 0.0 | 0.0 | 0.7 | 0.3 | 4.3 |
| **Lexington** | 0.0 | 0.1 | 0.2 | 0.5 | 0.2 | 3.7 |
| **Marion** | 0.1 | 0.0 | 0.1 | 0.7 | 0.0 | 3.4 |
| **Marlboro** | 0.4 | 0.1 | 0.0 | 0.1 | 0.3 | 2.7 |
| **McCormick** | 0.0 | 0.1 | 0.1 | 0.2 | 0.6 | 4.3 |
| **Newberry** | 0.0 | 0.1 | 0.1 | 0.5 | 0.2 | 3.8 |
| **Oconee** | 0.0 | 0.1 | 0.1 | 0.5 | 0.3 | 3.9 |
| **Orangeburg** | 0.1 | 0.1 | 0.1 | 0.4 | 0.3 | 3.6 |
| **Pickens** | 0.0 | 0.0 | 0.1 | 0.5 | 0.4 | 4.2 |
| **Richland** | 0.0 | 0.1 | 0.2 | 0.5 | 0.2 | 3.9 |
| **Saluda** | 0.0 | 0.0 | 0.7 | 0.0 | 0.3 | 3.7 |
| **Spartanburg** | 0.0 | 0.1 | 0.1 | 0.6 | 0.2 | 3.9 |
| **Sumter** | 0.0 | 0.2 | 0.2 | 0.5 | 0.1 | 3.4 |
| **Union** | 0.0 | 0.0 | 0.0 | 1.0 | 0.0 | 4.0 |
| **Williamsburg** | 0.0 | 0.1 | 0.2 | 0.5 | 0.2 | 3.9 |
| **York** | 0.0 | 0.1 | 0.2 | 0.5 | 0.2 | 3.8 |
| **MEDIAN** |  |  |  |  |  | 3.8 |

### Appendix 6 Garrett ranking analysis of SC residents’ preferred ecosystem services.

| Rank level | 1 | 2 | 3 | 4 | 5 | 6 | 7 | 8 |
| --- | --- | --- | --- | --- | --- | --- | --- | --- |
| Percent positions | 6.25 | 18.75 | 31.25 | 43.75 | 56.25 | 68.75 | 81.25 | 93.75 |
| Garrett Values | 80 | 67 | 60 | 53 | 47 | 40 | 32 | 20 |

| **Ecosystem Service** | **Frequency** | | | | | | | | **Overall Rank Score**  *(sum of Frequency of Rank_n*Garrett values of Rank_n)* | **Mean Value of Scores**  *(Overall rank scores / total respondents)* | **Overall Rank** |
| --- | --- | --- | --- | --- | --- | --- | --- | --- | --- | --- | --- |
|  | **Rank 1** | **Rank 2** | **Rank 3** | **Rank 4** | **Rank 5** | **Rank 6** | **Rank 7** | **Rank 8** |  |  |  |
| Water quality | 997 | 372 | 95 | 38 | 26 | 14 | 10 | 3 | 114560 | 73.91 | 1 |
| Water supply | 156 | 673 | 416 | 151 | 81 | 38 | 23 | 17 | 96937 | 62.54 | 2 |
| Air quality | 182 | 256 | 406 | 319 | 184 | 105 | 65 | 38 | 88667 | 57.20 | 3 |
| Wildlife and habitat conservation | 118 | 130 | 431 | 524 | 181 | 94 | 49 | 28 | 86177 | 55.60 | 4 |
| Tourism and recreation | 27 | 35 | 72 | 222 | 332 | 371 | 176 | 320 | 63067 | 40.69 | 5 |
| Heritage and cultural | 33 | 33 | 66 | 170 | 381 | 192 | 215 | 465 | 59588 | 38.44 | 6 |
| Hunting | 22 | 23 | 36 | 66 | 215 | 409 | 410 | 374 | 56024 | 36.14 | 7 |
| Fishing | 20 | 33 | 33 | 65 | 155 | 332 | 607 | 310 | 55425 | 35.76 | 8 |

### Appendix 7 Garrett ranking analysis of SC residents’ preferred ecosystems.

| Rank level | 1 | 2 | 3 | 4 | 5 | 6 | 7 |
| --- | --- | --- | --- | --- | --- | --- | --- |
| Percent positions | 7.14 | 21.43 | 35.71 | 50.00 | 64.29 | 78.57 | 92.86 |
| Garrett Values | 79 | 66 | 57 | 50 | 43 | 34 | 21 |

| **Type of Ecosystems** | **Frequency** | | | | | | | **Overall Rank Score**  *(sum of Frequency of Rank_n*Garrett values of Rank_n)* | **Mean Value of Scores**  *(Overall rank scores / total respondents)* | **Overall Rank** |
| --- | --- | --- | --- | --- | --- | --- | --- | --- | --- | --- |
|  | **Rank 1** | **Rank 2** | **Rank 3** | **Rank 4** | **Rank 5** | **Rank 6** | **Rank 7** |  |  |  |
| Forest | 377 | 386 | 317 | 244 | 144 | 61 | 26 | 94340 | 60.86 | 1 |
| Rivers/lakes | 316 | 315 | 248 | 226 | 280 | 134 | 36 | 88542 | 57.12 | 2 |
| Farm/agricultural land | 406 | 205 | 180 | 265 | 210 | 222 | 67 | 87099 | 56.19 | 3 |
| Wetland/marsh | 184 | 208 | 316 | 289 | 277 | 190 | 91 | 81008 | 52.26 | 4 |
| Mountain | 83 | 235 | 257 | 302 | 314 | 266 | 98 | 76420 | 49.30 | 5 |
| Coastal plains/beaches | 154 | 177 | 177 | 169 | 227 | 513 | 138 | 72488 | 46.77 | 6 |
| Hiking/biking trails | 35 | 29 | 60 | 60 | 103 | 169 | 1099 | 44353 | 28.61 | 7 |
